# Sequencing Targeted and Immune Therapy in *BRAF*-Mutant Melanoma: Lessons Learned

**DOI:** 10.1007/s11912-023-01402-8

**Published:** 2023-03-30

**Authors:** Claudia Trojaniello, Francesca Sparano, Eleonora Cioli, Paolo Antonio Ascierto

**Affiliations:** Melanoma, Cancer Immunotherapy, and Development Therapeutics Unit, Istituto Nazionale Tumori IRCCS Fondazione “G. Pascale”, Naples, Italy

**Keywords:** Melanoma, Sequence treatment, *BRAF*, Immunotherapy, Targeted therapy

## Abstract

**Purpose of Review:**

The treatment strategy for *BRAF*-mutated melanoma remains unsatisfactory, although the advent of immune checkpoint inhibition has improved the prognosis of advanced melanoma. This article reports current evidence on the efficacy and safety of sequential immunotherapy with targeted therapy in patients with *BRAF*-mutated melanoma. It discusses criteria for the use of available options in clinical practice.

**Recent Findings:**

Targeted therapy provides rapid disease control in a relatively high proportion of patients, although the development of secondary resistance limits the duration of responses; in contrast, immunotherapy may induce slow but more durable responses in a subset of patients.

**Summary:**

Therefore, the identification of a combination strategy for the use of these therapies seems a promising perspective. Currently, inconsistent data have been obtained, but most studies indicate that the administration of BRAFi/MEKi prior to immune checkpoint inhibitors appears to reduce the efficacy of immunotherapy. On the contrary, several clinical and real-life studies suggest that frontline immunotherapy with subsequent targeted therapy may be associated with better tumor control than immunotherapy alone. Larger clinical studies are ongoing to confirm the efficacy and safety of this sequencing strategy for treating *BRAF*-mutated melanoma with immunotherapy followed by targeted therapy.

## Introduction

The driver mutation of *BRAF* is found in approximately 50% of metastatic melanomas and represents a target for focused therapies in a population of patients with the aggressive disease [1]. Although the advent of an immune checkpoint inhibitor (ICI) and targeted therapy have dramatically improved the outcomes of advanced melanoma, *BRAF*-mutated patients continue to have a poor prognosis. Clinical trials showed that a single blockade of *BRAF* provided little benefit in terms of disease control because of the early occurrence of resistance. Although the addition of MEK inhibitors increased the time to progression, no advantage in survival was obtained toward combination immunotherapy [2, 3]. Indeed, the best treatment for patients with *BRAF*-mutated melanoma is still debated. Combination vs. sequencing immunotherapy with targeted therapy regimens is being investigated [4–6] to identify effective regimens for these patients.

This article reports current evidence on the efficacy and safety of sequential immunotherapy with targeted therapy in patients with *BRAF*-mutated melanoma. It discusses criteria for the use of available options in clinical practice.

## Immuno and Targeted Combination Therapy

Therapeutic options for *BRAF*-mutated melanoma in the first line include combinations of ICIs, nivolumab alone or with ipilimumab and pembrolizumab, or BRAF and MEK inhibitors (BRAFi/MEKi), dabrafenib and trametinib, encorafenib and binimetinib, and vemurafenib and cobimetinib [1].

Trials in advanced melanoma revealed that survival was higher with combined BRAF plus MEK inhibition during the first 6 months than with immunotherapy, after which ICIs provided a superior survival benefit [7]. Indeed, immunotherapy is characterized by a slow but durable response in several patients, while an early response is obtained in most *BRAF* V600-mutant patients with targeted therapy, but rapid onset of resistance is linked to a short duration of response (DOR). Therefore, combining targeted therapy with immunotherapy seems a rational approach to advanced melanoma, combining benefits from both treatments.

Preclinical and translational studies demonstrated an immunomodulatory effect mediated by BRAFi/MEKi on the tumor microenvironment [8], and phase I clinical trials explored the safety of BRAFi/MEKi combined with immunotherapy. The tolerability was poor for the combinations of vemurafenib with ipilimumab and dabrafenib with trametinib and ipilimumab, while better profiles were found combining BRAFi/MEKi with anti-PD-1 and anti-PD-L1 antibodies [9–11]. Additionally, good antitumor activity was demonstrated for the association of vemurafenib, cobimetinib, and atezolizumab, with an objective response rate (ORR) of 71.8% [11]. Another phase I study, the IMMU-TARGET trial, demonstrated the safety of encorafenib, binimetinib, and pembrolizumab, as well as an ORR response rate of 64% [12].

On these bases, several randomized clinical trials evaluated the efficacy of these combinations in the frontline setting for *BRAF*-mutated melanoma patients.

Three RCTs have investigated the association of BRAFi/MEKi with either anti-PD-1 or anti-PD-L1 antibodies and are now closed. The phase I/II clinical trial KEYNOTE-022 enrolled 120 subjects with unresectable locally advanced or metastatic *BRAF* V600E- or V600K-mutant melanoma who were randomly assigned to be treated with dabrafenib 150 mg BID and trametinib 2 mg daily combined with pembrolizumab 200 mg 3-weekly or placebo [13]. The phase III, IMspire150 or TRILOGY trial evaluated first-line vemurafenib and cobimetinib combined with either atezolizumab or placebo in 514 patients with unresectable stage IIIc–IV *BRAF* V600-mutated melanoma. Based on the phase I trial, the schedule consisted of a 28-day run-in of vemurafenib 960 mg BID and cobimetinib 60 mg daily from day 1 to 21, followed by a lower dose of vemurafenib (720 mg BID + vemurafenib or placebo) in the atezolizumab group. From the second cycle, atezolizumab 840 mg or placebo was added to these respective groups [14]. The COMBI-I, randomized phase III clinical trial, compared the combination of spartalizumab, dabrafenib 150 mg BID, and trametinib 2 mg daily versus dabrafenib, trametinib, and placebo in 532 subjects affected from advanced melanoma [15]. The median progression-free survival (PFS), the primary endpoint of the three RCTs, was 16.9 months in KEYNOTE-022, 15.1 in IMspire150, and 16.2 in COMBI-I in the triple therapy arms compared with 10.7, 10.6, and 12.0 months in the placebo arms, respectively [13–15]. However, only the IMspire150 trial demonstrated a statistically significant improvement in PFS (15.1 vs. 10.6 months) with a hazard ratio (HR) of 0.78 [14]. The secondary endpoints of the three trials were ORR, DOR, and overall survival (OS). Although the pembrolizumab arm of KEYNOTE-022 surprisingly showed a lower ORR (63%) than the control arm (72%), this finding was not replicated in any of the phase III trials. This could be due to the higher proportion of stage M1c patients in the triplet therapy arm of this study [13]. Importantly, the DOR was longer with triplet therapy across all three studies, supporting the hypothesis that the addition of ICI extends the longevity of response to BRAFi/MEKi. There was a trend to the improved OS in all three trials, although these data are still immature. Besides the good efficacy results, a high rate of adverse events (AEs) was observed in the triplet arms of all three trials (grade 3–5 AEs in the combination compared with the standard treatment arm of KEYNOTE-022 and COMBI-I were 58.3% vs. 25% and 54.7% vs. 33.3%, respectively) [13, 15]. Fever, rash, diarrhea, and liver transaminase elevation were reported more frequently for the combination groups. In the IMspire150 trial [14], even if the frequency of high-grade toxicities exceeded 70%, no difference was observed between the combination and placebo arms (79% and 73%, respectively); most events were asymptomatic and reversible laboratory abnormalities, with a low impact on treatment discontinuation (13% and 16% in the combination and placebo arms, respectively). In contrast, in the triple therapy arm of the KEYNOTE-022, AEs (mainly hepatitis or pneumonitis) caused treatment discontinuation in 47% of subjects compared with 20% in the control arm (Table 1) [13].

**Table 1:** Ongoing clinical trials exploring combination treatment with targeted therapy and immunotherapy in advanced BRAF-mutant melanoma

	KEYNOTE-022 (13)	IMspire 150 (16)	COMBI-I (15)
Subjects enrolled	120	514	532
PFS (months) Triplet vs. target	16.9 vs. 10.7	15.1 vs. 10.6 (HR 0.78)	16.2 vs. 12.0
ORR (%) Triplet vs. target	63.3 vs. 71.7	66.3 vs. 65.0	68.5 vs. 64.2
DOR (months) Triplet vs. target	25.1 vs. 12.1	21.0 vs. 12.6	NR vs. 20.7
OS at 24 months (%) Triplet vs. target	63 vs. 52	60.4 vs. 53.1	68 vs. 62
High grades toxicities (%) Triplet vs. target	58.3 vs. 25	79 vs. 73	54.7 vs. 33.3

Another study, the TRIdent phase II trial, tested nivolumab in combination with dabrafenib and trametinib in patients with *BRAF*-mutant and BRAFi/MEKi-naïve patients, including subjects refractory to immunotherapy and with brain metastases [16]. The incidence of treatment-related AEs was consistent with the results of the previous triplet therapy trials, but notably, only three patients discontinued due to toxicities. Overall, 27 patients received the triple therapy; 17 were PD-1 refractory, and 10 had brain metastases. ORR in 26 evaluable patients was 92% (three complete responses [CRs], 21 partial responses [PRs]). Among the progressing refractory patients evaluated for response, ORR was 88%; four of seven evaluated patients with brain metastasis achieved an intracranial response (57%), including two CRs. Median PFS for patients without brain metastasis was 8.5 months, 8.0 months for those with brain metastasis. Median OS was not reached [16]. An important limitation of all these trials is the absence of a comparison with ipilimumab and nivolumab, which represent the standard of care for unresectable melanoma [17]. Recently, the US FDA and EMA approved the combination of relatlimab and nivolumab, which is now available as an adjunctive immunotherapy option for this group of patients with minimal added toxicity [18].

After these trials demonstrated that the combination of BRAFi/MEKi/ICI could be an active treatment option for *BRAF*-mutated melanoma, further studies were carried out to identify candidate patients who may better benefit from this approach and the correct use of the new tools. The phase II TRICOTEL study showed that intracranial activity could be obtained by adding vemurafenib to cobimetinib in patients with *BRAF* V600-mutated melanoma [19]. It enrolled untreated melanoma patients with CNS metastases ≥ 5 mm and performance status ≤ 2, either *BRAF* V600 wild-type or *BRAF* V600 mutation-positive, who had completed adjuvant anti-PD-L1 therapy or were symptomatic and/or corticosteroid-depended. The *BRAF* V600-mutated subjects received atezolizumab (840 mg, days 1 and 15 of each 28-day cycle excluding the first one) plus oral vemurafenib (720 mg twice daily) plus oral cobimetinib (60 mg once daily, days 1–21). Intracranial ORR was 42% (95% CI: 29–54) in *BRAF* V600 mutation-positive subjects and 27% (95% CI: 8–55) in the *BRAF* V600 wild-type group. These results suggest that the triple therapy may reduce corticosteroid consumption and increase the benefit of atezolizumab [19].

## Sequential BRAF/MEK Inhibition and Checkpoint Blockade

As reported above, options for frontline therapy in progressed patients with *BRAF*-mutated melanoma include checkpoint inhibition and BRAF/MEK inhibition; however, no consensus has been reached on treatment choice and correct sequencing of therapy. Actually, clinicians consider patient and tumor-related criteria, such as the overall disease burden, LDH levels, and the presence of central nervous system (CNS) metastases [1]. In routine clinical practice, ICIs with a slow action and a low response rate with prolonged response duration are usually chosen for first-line treatment in asymptomatic patients [20–22]. However, we know that the best performance is expectedly in patients with good prognostic factors, even with the combination BRAFi/MEKi [23–25]. Due to early and high response rates with a short response duration, this approach is often used in an early phase in patients with symptomatic, bulky, and aggressive disease, high serum LDH concentrations, and ECOG performance status > 1, who need fast symptoms control [26–28]. More recently, the 2019 ESMO Clinical Practice Guidelines suggested that first-line therapy decisions need to be individualized according to the patient’s clinical status, comorbidities, treatment goals, and personal preferences. However, immunotherapy should still be preferred as first-line therapy for its durable disease control even after treatment discontinuation [29].

Besides choosing either of two main treatment strategies for patients with *BRAF*-mutant metastatic melanoma, clinical research addresses new questions regarding the appropriateness of combination or sequential use of immune and targeted therapy.

Some results from preclinical studies demonstrating an immunomodulatory effect of BRAFi/MEKi suggested that targeted therapy could enhance tumor susceptibility to ICIs by interfering with the microenvironment [8, 30]. In addition, observational data suggested that targeted therapy could decrease tumor burden and normalize LDH levels, resulting in enhanced ICIs efficacy [31]. On the contrary, some retrospective analyses provided evidence that pretreatment with BRAFi can have a negative effect on outcomes in patients subsequently treated with immunotherapy and reduce survival, possibly by inducing a resistant tumor microenvironment hostile to immunotherapy [32–34].

Recent evidence shows a clinical advantage for immunotherapy followed by targeted therapy.

The phase III randomized trial, DREAMseq (Doublet, Randomized Evaluation in Advanced Melanoma Sequencing) [4], compared the efficacy and toxicity of nivolumab/ipilimumab followed by dabrafenib/trametinib to the converse sequence progression in 265 patients with advanced *BRAF* V600-mutant melanoma. The patients were randomized to upfront dabrafenib plus trametinib (arm B) followed by ipilimumab plus nivolumab (arm D) at progression versus ipilimumab plus nivolumab (arm A) followed by dabrafenib plus trametinib (arm C) [35]. Enrollment in arms A and B represented step 1; in step 2, patients were enrolled in arms C and D. Data from this study showed that patients who received immunotherapy before targeted therapy had better outcomes than those treated with targeted therapy before immunotherapy. Specifically, at a median follow-up of 27.7 months, patients who first received ipilimumab plus nivolumab had a 2-year OS rate of 71.8% (95% CI: 62.5 − 79.1) vs. 51.5% (95% CI: 41.7 − 60.4) in those who first received dabrafenib plus trametinib, demonstrating a difference of 20.3% (95% CI: 2.6 − 37.9%; log-rank *p* = 0.010). The median PFS observed in arm A was 11.8 months (5.9 − 33.5 months) vs. 8.5 months (6.5 − 11.3 months) in arm B (log-rank *p* = 0.054); the 2-year PFS was 41.9% vs. 19.2%, respectively. The DOR was not reached in arm A (29.3 months–not reached) vs. 12.7 months (8.2 months − not reached) in arm B (log-rank *p* < 0.001). The ORR was similar in the first setting: 46% vs. 43% in arms A and B, respectively. After disease progression, the ORR was 47.8% vs. 29.6% in arms C and D, respectively. In step 2 of the study, the median PFS was 9.9 months (8.3 − 20.6 months) vs. 2.9 months (2.6 − 8.9 months), the ORR was 47.8% (26.8 − 69.4%) vs. 29.6% (12.7 − 47.2%), and the DOR was not reached (29.3 months − not reached) vs. 12.7 months (8.2 months − not reached) in arms C and D, respectively. The toxicity difference was insignificant between the treatment arms [35, 36]. Interestingly, study accrual was halted early because of a clinically meaningful difference in OS between treatment arms, allowing patients on the first-line targeted therapy to receive second-line combined ICIs without disease progression. OS rate at 3 years was 66.2% (56 − 74.6%) in arm A vs. 42.8% (32.9–52.4%) in arm B [36]. This trial reinforced the hypothesis that combined ICIs improve OS, producing durable responses, and are less effective if given after targeted therapy, whereas targeted therapy appears to work as well in the second-line setting as it does in the first line.

A Three-Arms Prospective, Randomized Phase II Study to Evaluate the Best Sequential Approach With Combo Immunotherapy (Ipilimumab/Nivolumab) and Combo Target Therapy (LGX818/MEK162) in Patients With Metastatic Melanoma and *BRAF* Mutation trial [5] is comparing upfront combined targeted therapy (encorafenib plus binimetinib) followed by combination immunotherapy (ipilimumab plus nivolumab) at disease progression (arm A) with upfront combination immunotherapy followed by combined targeted therapy at disease progression (arm B) in 209 patients with metastatic *BRAF* V600-mutant melanoma [37]. This trial will not only address the optimal sequencing of BRAFi/MEKi treatment and ipilimumab plus nivolumab until progression but will also include a third arm (arm C) to investigate the utility of a run-in phase of encorafenib–binimetinib of 8 weeks (sandwich arm), which may potentiate responses from subsequent ICI therapy before combined immunotherapy followed by combined targeted therapy. It is important to underline that the trial was not statistically powered to show a difference between the three arms and was designed as a non-comparative study. The primary endpoint is OS; secondary endpoints are PFS, total PFS, time to the second progression, percentage of patients alive at 2 − 3 years, best ORR, and DOR. At 32 months of follow-up, combination immunotherapy in the first line and sequential sandwich therapy showed better 2-year and 3-year OS trends than first-line targeted therapy (2-year OS: 73% vs. 69% vs. 65%; 3-year OS: 62% vs. 60% vs. 54%). The best ORR in the first line was 87%, 44.9%, and 82.4% in arms A, B, and C, respectively; however, in the second line, the best ORR was 25.7%, 57.9%, and 62.2%, respectively. Furthermore, patients treated in the first line with combined ICIs vs. second-line setting had improved ORR (45% vs. 26%). In addition, no benefit over combined ICIs followed by targeted therapy was shown in arm C [6].

Overall, such growing evidence from clinical trials promises a final understanding of the issue, whereas prior observations had provided inconsistent or low-grade evidence data, although mainly suggesting that immunotherapy after targeted therapy could have deleterious effects.

A clinical experience with 34 patients with metastatic melanoma [32] reported a median OS of5.7 months (95% CI: 5.0 − 6.3) in patients who received BRAFi before ipilimumab versus 18.6 months (95% CI: 3.2 − 41.3; *p* < 0.0001) in patients who completed ipilimumab before treatment with BRAFi; noteworthy, the patients initially treated with BRAFi were unable to complete the four cycles of ipilimumab therapy due to rapid progression. These data agreed with the results of patients treated according to the Italian expanded access program. Those who received BRAFi before immunotherapy had a median OS of 9.9 months versus 14.5 months of those who received ICIs before targeted therapy [33].

Another retrospective study reported poor outcomes for patients treated with ipilimumab following BRAFi, where only 50% of patients finished the four cycles of ipilimumab. In this study, the median PFS and OS from the start of BRAFi therapy were 6.7 months (95% CI: 4.3–9.1) and 19.6 months (95% CI: 10.0 − undefined), respectively, in patients who received BRAFi after immunotherapy, and 5.6 months (95% CI 4.7–6.8) and 13.4 months (95% CI: 10.1–17.0) in patients who received BRAFi before immunotherapy [38].

Simeone et al. reported the outcomes of 47 patients (42 analyzed) with metastatic cutaneous, mucosal or ocular melanoma treated with pembrolizumab after progression or unacceptable toxicity on ipilimumab as part of the Italian expanded access program [39]. In total, 16 of them had *BRAF* V600-mutated melanoma and had been previously treated with vemurafenib or dabrafenib. These latter patients had a lower median PFS (3 months vs. not reached; *p* = 0.001) and disease control rate (DCR; 18.6% versus 65.4%; *p* = 0.005) than patients with *BRAF* wild-type; the response rate was also lower, although not significantly (12.5% versus 36.4%; *p* = 0.16).

Treatment with pembrolizumab showed a reduced ORR in patients previously treated with a BRAFi compared with the population in the KEYNOTE-001 study, suggesting that prior therapy with a BRAFi could reduce the response to ICIs [40]. In the phase II KEYNOTE-002 study, patients with ipilimumab-refractory melanoma were randomized to pembrolizumab or investigator choice chemotherapy, and the patients with *BRAF* V600-mutated were treated with BRAF inhibitor. In the pembrolizumab arms, the median PFS was 3.8 months in *BRAF* wild-type melanoma patients versus 2.8 months in *BRAF*-mutant melanoma patients; the 6-month PFS rate was 40.9% versus 19.5%, with an ORR of 26.7% vs. 11.9%, respectively [41]. In the phase III KEYNOTE-006 trial, in which patients with *BRAF* wild-type and *BRAF*-mutant melanoma were randomized to first-line pembrolizumab or ipilimumab, among patients with *BRAF*-mutant melanoma, those who had not been treated previously with a BRAFi had longer mPFS than patients who had received previous BRAFi treatment (7.0 vs. 2.8 months; 6-month PFS rate: 52.7% vs. 32.2%) [42].

Nevertheless, evaluating the DOR to BRAFi/MEKi and how it affects subsequent ICI therapy is important to further understand the relationship between the two treatments. Indeed, Da Silva et al. reported that the ORR to subsequent anti-PD-1 therapy was 34% in patients who responded to BRAF/MEK inhibition for more than 6 months, and 15% in those who responded to BRAF/MEK inhibition for ≤ 6 months [43].

On the other hand, some data suggested that prior exposure to anti-PD-1 therapy might negatively impact the response to subsequent BRAFi therapy. Recently, concern has been raised about the tolerability and side effect profile of BRAFi/MEKi after anti-PD-1 therapy [44]. In a retrospective analysis of 114 patients with advanced *BRAF*-mutant melanoma, the median OS was similar in those who had the anti-PD-1 in the first line and those who received BRAF ± MEK inhibitors before the anti-PD-1 (27.5 vs. 40.3 months; *p* = 0.71) [45]. Patients who progressed on anti-PD-1 during the study had worse outcomes after starting subsequent BRAFi than those who had not received prior anti-PD-1 (median PFS [mPFS] 5 vs. 7.4 months, median OS [mOS] 10.6 vs. 40.3 months). Patients who previously progressed with BRAFi treatment had inferior outcomes after starting anti-PD-1 compared with those without prior BRAFi, including ORR (25% vs. 41%), mPFS (2.8 vs. 10.6 months), and mOS (8.2 vs. 27.6 months). Patients who received at least 6 months of BRAF inhibitor therapy had superior ORR to subsequent anti-PD-1 therapy compared with those with more rapid progression (< 6 months) on BRAFi (34% versus 15%, *p* = 0.04), suggesting that this negative impact was a reflection of baseline tumor biology rather than an impact of the BRAFi therapy on the tumor immune microenvironment.

Other data suggested that first-line immunotherapy does not negatively impact the prognosis of patients receiving subsequent BRAFi and may be associated with improved OS compared to patients initially treated with BRAFi/MEKi [46, 47]. Moser et al. found that frontline treatment with PD-1 and nivolumab/ipilimumab were associated with statistically longer survival than BRAFi/MEKi in multivariate analyses, with a 34% and 49% mortality risk reduction in patients who received first-line nivolumab plus ipilimumab versus targeted therapy and first-line anti-PD-1 alone versus targeted therapy, respectively [47]. In a retrospective trial, 79 patients with *BRAF*-mutant metastatic melanoma were treated with ≥ 1 line of immunotherapy followed by subsequent BRAFi/MEKi [48]. The median PFS was 4.4 months, the median OS from the start of BRAFi/MEKi treatment was 18.0 months, and 39% were alive at 3 years, suggesting that BRAF/MEK inhibition is effective in patients with *BRAF* V600-mutant melanoma previously treated with immunotherapy with response rates similar to those seen in trials of first-line treatment with BRAF/MEK inhibition.

Haist et al. demonstrated in a retrospective real-world cohort study that in 135 *BRAF*-mutant melanoma patients, frontline ICI therapy is associated with favorable tumor control and OS vs. frontline targeted therapy (OSm 35 vs. 18 months, *p* = 0.07), although the difference was not statistically significant (significance was reached in a subgroup of patients without previous systemic treatments, with median OS 41 vs. 14 months, *p* = 0.02) [49]. Furthermore, this study showed a better ORR to second-line therapy for patients receiving first-line ICI (18.4% vs. 37.8%, *p* = 0.024) with a high risk of rapid progression in patients who were refractory to first-line BRAF/MEK inhibition (27.6% vs. 16.2%) [49].

It should also be mentioned that ICI combination therapy has been associated with sustained clinical benefit beyond treatment discontinuation, delaying subsequent treatment initiation and resulting in long treatment-free intervals, which favors the use of sequencing treatments [50–52]. Pooled data from the CheckMate 067 and CheckMate 069 trials showed a longer treatment-free interval in patients on nivolumab + ipilimumab compared with those on nivolumab and ipilimumab alone [52]. In a further analysis, estimated survival gain was higher for sequences initiating with anti-PD-1 + anti-CTLA-4 than for anti-PD-1 monotherapy or BRAFi + MEKi [53]. This analysis showed approximately 5 additional total life-years for patients who received first-line anti-PD-1 + anti-CTLA-4 (8.4 years) compared with first-line BRAFi/MEKi (3.2 years); furthermore, treatment with BRAFi/MEKi following ICI therapy (anti-PD-1 or anti-PD-1 + anti-CTLA-4) provided numerically higher life-year benefit as with first-line BRAFi/MEKi (1.3 vs. 1.1 years, respectively). Treatment with anti-PD-1 + anti-CTLA-4 followed by subsequent BRAFi/MEKi was also associated with the longest gain in total quality-adjusted life-years: 6.5 years with first-line anti-PD-1 + anti-CTLA-4, 5.4 years with first-line anti-PD-1, and 2.6 years with first-line BRAFi/MEKi.

Additional evidence supporting sequencing therapy comes from a real-world analysis in which patients treated with first-line nivolumab + ipilimumab showed significant survival benefits versus those receiving first-line BRAFi/MEKi [54]. Specifically, nivolumab + ipilimumab was associated with a 32% reduction in risk of death compared with patients who received BRAFi/MEKi. At a mean follow-up of 15–16 months, 64% of nivolumab + ipilimumab patients and 43% of BRAFi/MEKi patients were alive. After first-line nivolumab + ipilimumab, 20% of patients died before subsequent therapy, whereas 32% died after first-line BRAFi/MEKi.

An observational study evaluated more than 1000 patients with metastatic melanoma with *BRAF* V600 mutation and treated in the first line with either BRAFi/MEKi or ICIs (PD-1 single agent or combined PD-1/CTLA-4 antibodies), included in the EUMelaReg treatment registry [55]. Primary endpoints were OS and second-line PFS (PFS-2, defined as the interval from the start of first-line therapy to a progression after a second-line treatment or death of any cause). The ORR for BRAFi/MEKi was significantly higher than for ICI (53.3% vs. 42.0%; *p* = 0.0004), but for OS and PFS2, the adjusted HR was better for ICI (HR 0.62 and 0.66, respectively; *p* < 0.0001). In the second line, patients switching from ICI to BRAFi/MEKi had important higher ORR than patients switching from BRAFi/MEKi to ICI (57.7% vs. 19.9%; *p* < 0.0001) and significantly longer unadjusted PFS (8.1 vs. 3.1 months; *p* < 0.0001) and OS (15.7 vs. 10.6 months; *p* = 0.01) after the start of second-line treatment. First-line ICI still resulted in significantly longer OS than BRAFi/MEKi after adjusting the analysis for imbalances, including the number of metastatic sites, AJCC substage, serum LDH, and ECOG performance status (Table 2) [55].

**Table 2:** Factors favoring ICIs vs. TT

	ICIs	Target therapy
Rate of objective response	Lower	Higher
Onset of action	Slower	Faster
Profound response	Higher	Lower
Level of disease control	Higher	Lower
Duration of response	Higher	Lower
Control of disease even after treatment discontinuation	Yes	Not
Grade 3–5 toxicities		Fewer
Reversible toxicities		Spesso
Treat patients with autoimmune conditions and on immunosuppressive therapies	To evaluate	Yes

### Ongoing Trials

Ongoing trials are further investigating sequencing strategies for immunotherapy and targeted therapy in *BRAF* V600-mutant melanoma to evaluate specific regimens.

Combination targeted therapy (encorafenib and binimetinib) followed by combination immunotherapy (ipilimumab and nivolumab) is also being investigated in 270 patients with unresectable or metastatic melanoma with *BRAF* V600 mutation in the randomized, multicentre, comparative phase II Combination of Targeted Therapy (Encorafenib and Binimetinib) Followed by Combination of Immunotherapy (Ipilimumab and Nivolumab) vs. Immediate Combination of Immunotherapy in Patients With Unresectable or Metastatic Melanoma With *BRAF* V600 Mutation: an EORTC Randomized Phase II Study (EBIN) study [56]. Patients will be randomized to upfront ipilimumab plus nivolumab for 12 weeks, followed by nivolumab monotherapy for up to 2 years or until disease progression, compared with immediate encorafenib and binimetinib for 12 weeks, followed by ipilimumab plus nivolumab for another 12 weeks, nivolumab monotherapy for up to 2 years or until disease progression, and then encorafenib and binimetinib until disease progression. This trial is based on preclinical data supporting the rationale for intermittent regimens with BRAFi, showing that the development of resistance could be delayed. Targeted therapy might sensitize the tumor cells to immune attacks by increasing antigen expression and enhancing immune cell effector function. Therefore, a sequential approach could combine the high response rate of targeted therapy with the peculiarity of immunotherapy to achieve long-term durable responses before the initiation of secondary resistance to the targeted therapy. The primary endpoint is PFS; secondary endpoints are OS, CR, PDR, and safety. The first results from the trial are expected around mid-2023.

Similarly, a phase II trial (A Phase II, Open-label, Randomized-controlled Trial Evaluating the Efficacy and Safety of a Sequencing Schedule of Cobimetinib Plus Vemurafenib Followed by Immunotherapy With an Anti-PD-L1 Antibody Atezolizumab for the Treatment in Patients With Unresectable or Metastatic BRAF V600 Mutant Melanoma) is evaluating, in 176 patients with unresectable or metastatic *BRAF*-V600 mutant melanoma, the safety and efficacy of atezolizumab (arm A) or vemurafenib and cobimetinib (arm B) after a 3-month run-in period with vemurafenib plus cobimetinib [57]. Interim analysis at a median follow-up of 19 months showed significantly longer median PFS1 (time from start of the run-in to first progression or death) in arm A vs. B (HR 0.55; 95% CI: 0.37–0.84; *p* = 0.001), a not significantly different median PFS2 (time from the start of the run-in to second progression or death) between arms (HR 1.57; 95% CI: 0.83–2.96; *p* = 0.163) and a shorter median PFS3 (time from the first progression to second progression or death) in arm A vs. B (HR 2.24; 95% CI: 1.17–4.30; *p* = 0.013). OS was similar between arms (HR: 1.22; 95% CI: 0.69–2.16; *p* = 0.389). Grade 3/4 AEs occurred in 55% of patients in arm A and 64% in arm B; AEs led to discontinuation in 10% and 12%, respectively [58]. Thus, preliminary results suggest that a planned switch to atezolizumab after 3 months of treatment with cobimetinib + vemurafenib did not prolong PFS and OS but is feasible and safe in patients with *BRAF* V600-mutated melanoma.

Finally, a phase II trial (Phase 2 Study With COmbination of Vemurafenib With Cobimetinib in B-RAF V600E/K Mutated Melanoma Patients to Normalize LDH and Optimize Nivolumab and Ipilimumab therapY, COWBOY) is studying the efficacy of 6-week induction therapy with vemurafenib + cobimetinib, followed by ipilimumab plus nivolumab, vs. upfront ipilimumab plus nivolumab in 83 patients with metastatic *BRAF* V600 melanoma [59]. This study is aimed at investigating whether the pre-administration of vemurafenib and cobimetinib may normalize LDH levels, which appear to be associated with tissue damage and rapid growth of melanoma, resulting in improved ICI efficacy (Table 3).

**Table 3:** Ongoing clinical trials exploring sequence treatment with targeted therapy and immunotherapy in advanced BRAF-mutant melanoma

Study name NCT number	Phase	No. of patients	Study design	Primary endpoint	Outcomes
DREAMSeq (54, 55) NCT02224781	III Randomized, open-label	265	**Arm A**: ipilimumab/nivolumab q3w for 2 cycles, followed by Nivo monotherapy and subsequent dabrafenib/trametinib at PD** (ARM C)** **Arm B**: dabrafenib/trametinib followed by ipilimumab/nivolumab at PD q3w for 2 cycles, followed by Nivo monotherapy (**ARM D)**	2-year OS	2-year OS = arm A: 71.8%, arm B:51.5% mPFS = arm A: 11.8 m, arm B: 8.5 m, arm C: 9.9 m, arm D: 2.9 m DOR = arm A: NR, arm B: 12.7 m, arm C: NR, arm D: 12.7 m ORR = arm A: 46%, arm B: 43%, arm C: 47.8%, arm D: 29.6% OS 3 years = arm A: 66.2%, arm B 42.8%
SECOMBIT (6) NCT02631447	II Randomized, open-label	209	**Arm A**: encorafenib/binimetinib followed by ipilimumab/nivolumab q3w for 4 cycles and subsequent Nivo q2w at PD **Arm B**: ipilimumab/nivolumab q3w for 4 cycles and subsequent Nivo q2w followed by encorafenib/binimetinib at PD **Arm C**: encorafenib/binimetinib run-in (8 weeks) then ipilimumab/nivolumab q3w for 4 cycles and subsequent Nivo q2w, followed by encorafenib/binimetinib at PD	OS	2-year OS = arm A: 65%, arm B: 73%, arm C: 69% 2 yr PFS = arm A: 46%, arm B: 65%, arm C: 57% 3-yr OS = arm A:54%, arm B:62%, arm C: 60%
ImmunoCobiVem (59) NCT02902029	II Randomized, open-label	176	**Arm A**. run-in vemurafenib/cobimetinib (3 months) then vemurafenib/cobimetinib followed by atezolizumab 1200 mg q3w at PD **Arm B**. run-in vemurafenib/cobimetinib (3 months) then atezolizumab 1200 mg q3w followed by vemurafenib/cobimetinib at PD	Time to PFS2	PFS1 = arm A: 13.9 m, arm B: 5.9 m PFS2 = arm A: 12.6 m, arm B: 14.9 m PFS3 = arm A: 2.8 m, arm B: 6 m
EORTC 1612 (57) (EBIN) NCT03235245	II Randomized, open-label	270	**Arm A**: ipilimumab/nivolumab q3w followed by Nivo q4w until completion of 2 years or PD **Arm B**: encorafenib/binimetinib run-in (12 wks) then ipilimumab/nivolumab q3w followed by Nivo q4w until completion of 2 years or PD followed by encorafenib/binimetinib at PD	mPFS	Recruiting
COWBOY (60) NCT02968303	II Randomized, open-label	83	**Arm A.** 6 weeks of Vem + Cob followed by IPI + Nivo and Nivo q4w **Arm B.** Upfront IPI + Nivo followed by Nivo q4w, without prior induction with Vem + Cob	BORR	Recruiting

## Conclusion

Available evidence shows that BRAFi/MEKi may provide rapid disease control in a relatively high proportion of patients with *BRAF*-mutated melanoma, although the development of secondary resistance limits the DORs. Besides, ICIs may induce slow but more durable responses in a subset of patients with both *BRAF*-mutant and wild-type melanoma. Identification of a combination strategy for using these therapies seems a promising perspective for this setting, which has a poor prognosis even after the improvements brought about by the advent of immunotherapy on outcomes for the overall population of patients with advanced melanoma. Currently, inconsistent data have been obtained, but most studies indicate that the administration of BRAFi/MEKi prior to ICIs appears to reduce the efficacy of immunotherapy. In contrast, several clinical and real-life studies suggest that frontline immunotherapy with subsequent targeted therapy may be associated with better tumor control than immunotherapy alone. Larger clinical studies are ongoing to confirm the efficacy and safety of this sequencing strategy for treating *BRAF*-mutated melanoma with immunotherapy followed by targeted therapy.
